# Prognostic Immune-Related Analysis Based on Differentially Expressed Genes in Left- and Right-Sided Colon Adenocarcinoma

**DOI:** 10.3389/fonc.2021.640196

**Published:** 2021-03-08

**Authors:** Jun-Nan Guo, Ming-Qi Li, Shen-Hui Deng, Chen Chen, Yin Ni, Bin-Bin Cui, Yan-Long Liu

**Affiliations:** ^1^ Department of Colorectal Surgery, Harbin Medical University Cancer Hospital, Harbin, China; ^2^ Department of Anesthesiology, The Fourth Affiliated Hospital of Harbin Medical University, Harbin, China

**Keywords:** colon adenocarcinoma (COAD), left-sided, right-sided, immune-related genes (IRGs), prognosis

## Abstract

**Background:**

Colon adenocarcinoma (COAD) can be divided into left-sided and right-sided COAD (LCCs and RCCs, respectively). They have unique characteristics in various biological aspects, particularly immune invasion and prognosis. The purpose of our study was to develop a prognostic risk scoring model (PRSM) based on differentially expressed immune-related genes (IRGs) between LCCs and RCCs, therefore the prognostic key IRGs could be identified.

**Methods:**

The gene sets and clinical information of COAD patients were derived from TCGA and GEO databases. The comparison of differentially expressed genes (DEGs) of LCCs and RCCs were conducted with appliance of “Limma” analysis. The establishment about co-expression modules of DEGs related with immune score was conducted by weighted gene co-expression network analysis (WGCNA). Furthermore, we screened the module genes and completed construction of gene pairs. The analysis of the prognosis and the establishment of PRSM were performed with univariate- and lasso-Cox regression. We employed the PRSM in the model group and verification group for the purpose of risk group assignment and PRSM accuracy verification. Finally, the identification of the prognostic key IRGs was guaranteed by the adoption of functional enrichment, “DisNor” and protein-protein interaction (PPI).

**Results:**

A total of 215 genes were screened out by differential expression analysis and WGCNA. A PRSM with 16 immune-related gene pairs (IRGPs) was established upon the genes pairing. Furthermore, we confirmed that the risk score was an independent factor for survival by univariate- and multivariate-Cox regression. The prognosis of high-risk group in model group (P < 0.001) and validation group (P = 0.014) was significantly worse than that in low-risk group. Treg cells (P < 0.001) and macrophage M0 (P = 0.015) were highly expressed in the high-risk group. The functional analysis indicated that there was significant up-regulation with regard of lymphocyte and cytokine related terms in low-risk group. Finally, we identified five prognostic key IRGs associated with better prognosis through PPI and prognostic analysis, including IL2RB, TRIM22, CIITA, CXCL13, and CXCR6.

**Conclusion:**

Through the analysis and screening of the DEGs between LCCs and RCCs, we constructed a PRSM which could predicate prognosis of LCCs and RCCs, and five prognostic key IRGs were identified as well. Therefore, the basis for identifying the benefits of immunotherapy and immunomodulatory was built.

## Introduction

With year-by-year increase of colorectal cancer (CRC) incidence worldwide, CRC is considered as one of the main causes of death due to cancer (1). As the complexity of physiology and anatomy, the distinction of CRCs can be performed in accordance with their primary tumor location in colon and rectum. Cancers located in the colon can be divided into left-sided colon cancers (LCCs) and right-sided colon cancers (RCCs) as per different definitions (2–4). Although the CRCs are mainly distinguished with embryonic origin, there are great significances existing between the LCCs and RCCs in various clinical aspects, such as metastasis tendency, survival and prognosis, chemotherapy drugs, immunotherapy, and sensitivity of molecularly targeted drugs, etc. (5–7). The difference in prognosis makes colon cancer sidedness a criterion for predicting prognosis of all clinical stages (8). These differences have also given us incentives to gain deep understanding of the molecular biological mechanism.

Recent studies have analyzed the differences between LCCs and RCCs from different perspectives, including embryonic origin, microbes, chromosomal and molecular, blood vessel supply, and physiological functions, etc. (9). Generally, these studies indicated the reasons for the differences in the sensitivity of chemotherapy and molecular targeted drugs (10). Therefore, it is very necessary for us to take the CRCs locations into full consideration upon determining treatment options (8).

Concerning the researches of various cancers, as one of the important components of the TME, the tumor-associated immune microenvironment (TAIM) is driving force for the heterogeneity, plasticity, and evolution of tumors (11). Over recent years, immunotherapy has gradually become the primary direction of future tumor treatment development due to its minimal side effects and obvious effects. Immunotherapy is the fourth most frequently applied tumor treatment technology after surgery, radiotherapy, and chemotherapy (12). The study on TAIM differences between LCCs and RCCs has shown great potential in terms of accurate prognostic biomarkers finding and patient prognosis prediction, as well as the identification of the greatest therapeutic benefit. In the meantime, it has provided a molecular basis for the improvement of immunotherapy by TAIM regulation.

In this study, we analyzed the genes differentially expressed in the LCCs and RCCs in The Cancer Genome Atlas (TCGA) database. We used weighted gene co-expression network analysis (WGCNA) to select the module genes with the highest correlation with immune score, so as to construct immune-related gene pairs (IRGPs). Furthermore, a prognostic risk scoring model (PRSM) was established by the IRGPs. The PRSM, which was verified in the Gene Expression Omnibus (GEO) database, calculated the risk score (RS) of patients, and divided them into high- and low-risk group (HRG and LRG, respectively) with poor diagnosis. Finally, we identified prognostic key immune-related genes (IRGs).

## Materials and Methods

### Colon Cancer Samples From TCGA and GEO Databases

In this study, we adopted two independent gene data-sets from different high-throughput platforms, including 473 COAD samples from TCGA and 156 COAD samples from GEO (GSE103479) respectively. In accordance with the downloaded clinical information, gene expression data, and corresponding overall survival information of the LCCs and RCCs were screened out. The CRCs in cecum, ascending colon and hepatic flexure were defined as LCCs. The CRCs in plenic flexure, descending colon, sigmoid colon, and rectosigmoid junction were defined as RCCs. There were a total of 411 samples with complete information available for analysis, in which 322 from TCGA and 89 from GEO. The above analysis excluded that RNA was undetectable in more than 10% of the samples. Concerning each data-set, the gene ID was converted to gene symbol in accordance with the corresponding annotation package. We chose the TCGA data as the model group, and GEO data as the verification group.

### Identification of Differential Gene Consensus Modules and Correlation Analysis With Immune Score

We used the R package “Limma” to analyze the differentially expressed genes (DEGs) in LCCs and RCCs from TCGA (|log2foldchange|>0.5, P-adj<0.05) (13). The estimation of the stromal cells and immune cells in LCCs and RCCs tissues was conducted by R package “ESTIMATE” (14). The “ESTIMATE” package is a tool based on the ssGSEA method to rate tumor expression matrix in accordance with stromal and immune gene sets.

For the purpose of analyzing of gene expression landscape concerned with immune cell infiltration score, we employed the DEGs for WGCNA to identify consensus gene modules by the R package “WGCNA” (15). To start with, we constructed the adjacency matrix (AM) of paired genes by power function. An appropriate power index was selected so as to increase the similarity of matrix and achieve a scale-free co-expression network. Then AM was converted into a topological overlap matrix (TOM). We used TOM based on dissimilarity measurements to perform average linkage hierarchical cluster analysis. Finally, we obtained gene dendrogram and gene consensus modules. Module eigengenes (MEs) were defined as the main components of each module. For obtaining the correlation coefficient (CC), the analysis of MEs was performed by the stromal and immune scores respectively. Gene significance (GS) was identified as mediated p-value of each gene (GS = lgP) in the linear regression between gene expression and the scores.

### Further Screening of Immune-Related Genes and Construction of IRGPs

We selected the module with the highest correlation with immune score, and then calculated the GS and module membership (MM) of each gene. Module membership is a measure of intra-modular connectivity. In order to avoid missing IRGs, we defined the screening threshold as Cor. gene MM>0.5 and Cor. gene GS>0.5. To eliminate the measurement error of gene expression between different samples, we constructed the IRGs into gene pairs. That is to say, we compared the expression levels of two genes in the same sample. If the former gene was greater than the latter gene, the output was 1, otherwise the output was 0. After we removed IRGPs with small variation and unbalanced distribution (MAD = 0), remaining IRGPs were constructed by using univariate Cox proportional hazards regression analysis. The IRGPs with p<0.05 in Cox regression were retained for lasso-Cox proportional hazards regression with 1000 simulations by the R package “glmnet” (16). The immune-related features of IRGPs in PRSM was obtained from The Human Gene Database (https://www.genecards.org). Time dependent receiver operating characteristics (timeROC) for 3, 5, 10 years were plotted in the model group by the R package “survivalROC” (17). The best cut-off value of risk score (RS) was determined by ROC curve at appropriate period of time. Finally, with the application of PRSM in the model group and validation group, therefore patients could be divided into HRG and LRG with poor prognosis.

### Validation of the Predictive Model

With the adoption of long-rank test, we analyzed the prognosis of patients with high- and low-risk in the model group and validation group. The purpose was to verify the predictive effect of PRSM in grouping. Then, after the combination with other clinical factors, we used univariate- and multivariate-Cox proportional hazard analysis to verify the independent predictive effect of RS.

### Immune Infiltration in HRG and LRG

In order to specifically analyze the differences of immune infiltration in the HRG and LRG, we adopted R package “CIBERSORT” (18) to evaluate the relative infiltration abundance of 22 types of immune cells in each sample. “CIBERSORT” calculated the p value of the deconvolution for each sample by Monte-Carlo simulation to provide the estimated confidence. Then, we reserved the samples with p<0.05 estimated by “CIBERSORT,” and analyzed the difference of 22 types of immune cells in HRG and LRG by Wilcoxon rank sum test. Finally, the difference of immune infiltration in HRG and LRG was obtained.

### Gene Ontology, Kyoto Encyclopedia of Genes and Genomes, and Gene Set Enrichment Analysis

For the purpose of studying the biological functions of differential IRGs and genes in PRSM, we employed R package “clusterProfiler” (19) to perform GO functional annotations and KEGG pathway enrichment analysis. In order to compare the gene sets between HRG and LRG, the ratio of the gene expression was converted by log2 fold change and ranked. The GSEA was carried out by adopting the Bioconductor package “fgsea” (20) with 10,000 permutations. The threshold values were p<0.01 and FDR<0.05.

### Identification of Key Prognostic Immune-Related Genes

We performed protein-protein interaction (PPI) analysis by STRING (https://www.string-db.org) on the IRGs screened in the selected module, so as to identify the key prognostic IRGs. We selected the genes with more than 10 interaction nodes in the network to intersect with IRGPs genes in PRSM. The survival curves were plotted and the differences were analyzed. Furthermore, the R package “maxstat” (21) was performed to get the cut-off value. The Kaplan-Meier method was used for the visualization purposes, and we calculated the differences between survival curves with log-rank test by R packages “survcomp” (22). Finally, we used DisNor (https://disnor.uniroma2.it/) to analyze the upstream genes, downstream genes and protein interactions of key prognostic IRGs. DisNor is a disease-focused resource that adopts the causal interaction information annotated in SIGNOR and the protein interaction data in Mentha to generate and explore protein interaction networks linking disease genes.

## Results

### DEGs and Immune Score in LCCs and RCCs

**Figure 1:**
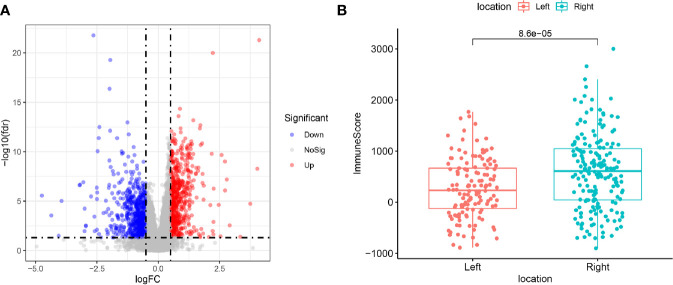
**(A)** Differentially expressed genes between left-sided colon cancers (LCCs) and right-sided colon cancers (RCCs). Red and blue circles indicate high and low genes expression, respectively. **(B)** Differences in immune score between LCCs and RCCs. LCCs and RCCs, left and right-sided colon adenocarcinoma.

For the aim of studying the DEGs between LCCs and RCCs, TCGA data were filtered, grouped, normalized, and differential expression analyzed. Through these processes, 1,327 DEGs were screened out (**Figure 1A**). We preformed the “ESTIMATE” to estimate the immune score and stromal score of these samples. It was found that there was a significant difference in immune score between LCCs and RCCs (**Figure 1B**).

### Screening of the Most Significant Modules and Immune-Related Genes by WGCNA

**Figure 2:**
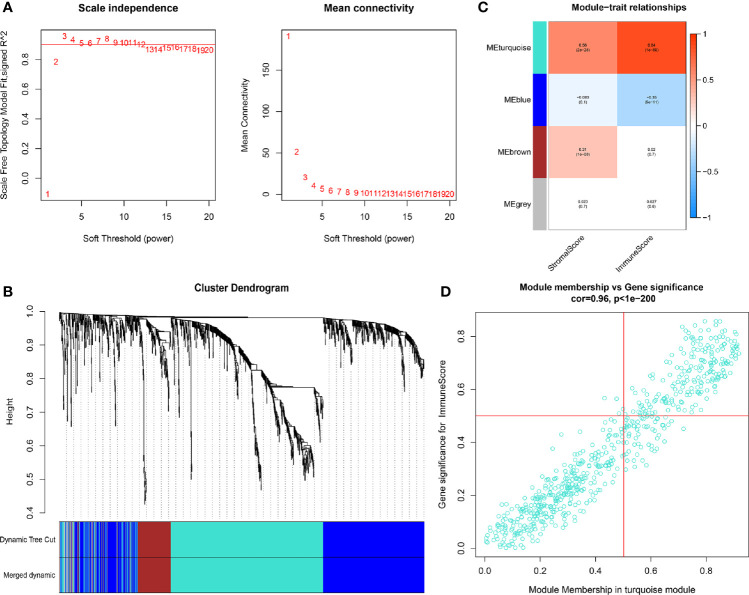
**(A)** In order to achieve a scale-free co-expression network, we chose power index = 3 as the appropriate soft threshold. **(B)** Identification of a co-expression module. The branches of the dendrogram correspond to four different gene modules. **(C)** Correlation between the gene modules and tumor microenvironment related scores, including immune score and stromal score. Each cell contains corresponding correlation coefficient and *p*-value. The correlation coefficient decreased in size from red to blue. **(D)** Scatter plot of module eigengenes in the turquoise module.

With the use of WGCNA, we constructed the gene co-expression network to identify biologically important gene modules, so as to have further understanding of the genes causing the differences of immune infiltration between LCCs and RCCs. After the removal of outlier samples, we chose power index which is equal to 3 as the soft threshold (scale-free R2 = 0.956) (**Figure 2A**). A scale-free co-expression network was constructed by using 1,327 DEGs (**Figure 2B**). Finally, four modules, CC and p values were obtained (**Figure 2C**). We figured out that turquoise module had the highest correlation with immune score (CC=0.84, p <0.001). Therefore, we chose the turquoise module for subsequent analysis.

### Construction of PRSM using IRGPs

**Figure 3:**
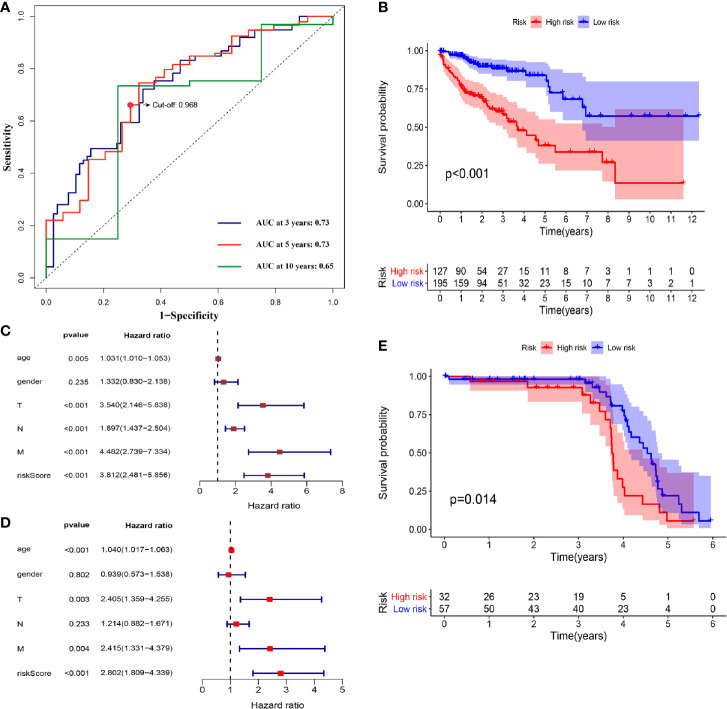
**(A)** TimeROC curves for 3, 5, 10 years were plotted in the model group. The best cutoff value was marked on the 5-year TimeROC curve. **(B)** Kaplan-Meier curve of overall survival in model group. **(C)** Univariate-Cox regression analyze of prognostic factors in model group. **(D)** Multivariate-Cox regression analyze of prognostic factors in model group. **(E)** Kaplan-Meier curve of overall survival in validation group.

We further screened 215 relatively critical IRGs (cor. gene MM>0.5 and cor. gene GS>0.5) (**Figure 2D**). The establishment of 23,005 IRGPs was conducted by pairwise alignment of these 215 genes. After the removal of the IRGPs with small variation (0 or 1< 20%), the remaining 809 IRGPs were analyzed by univariate-Cox proportional hazards regression. There were significant differences in 69 IRGPs (p< 0.05) (**Supplementary Table 1**). Then, we preformed the analysis of these IRGPs in the model group by using lasso-Cox proportional hazards regression. In the final PRSM, 16 prognostic-related IRGPs and corresponding risk coefficients were determined (**Table 1**). The RS of each patient in the model group was calculated by the PRSM. We adopted TimeROC in different time periods, it was found that the area under curve (AUC) of 3 and 5 years were the highest (all AUC=0.73). Based on the 5-year ROC curve, we set the best cutoff value as 0.968 to classify the patients into HRG and LRG (**Figure 3A**, **Supplementary Table 2**). A survival curve was plotted for patients in the HRG and LRG, and the result showed that the OS of HRG was significantly lower than that of LRG (p<0.001) (**Figure 3B**). Then, we processed univariate- and multivariate-Cox regression by the combination of RS with clinical information. The result indicated that the RS was an independent factor affecting the prognosis (**Figures 3C, D** **)**.

**Table 1:** Prognostic risk scoring model (PRSM) information including 16 immune-related gene pairs (IRGPs).

IRG 1	Immune-related features	IRG 2	Immune-related features	Coefficient
APBB1IP	Cytokine signaling	SLA	T-cell receptor signaling	0.36
SIGLEC10	Immunoglobulin	KLRB1	Lectin-like receptor	0.24
NFAM1	Type-I membrane receptor	SLA	T-cell receptor signaling	0.22
FAM78A	Protein binding	SIRPG	Immunoglobulin-like cell surface receptor	0.12
FAM78A	Protein binding	CIITA	Interferon gamma signaling	0.33
IL2RB	Cytokine receptors	ODF3B	Protein binding	-0.46
DOCK2	Chemokine Signaling	CXCR6	Chemokine receptor	0.20
ARHGAP25	Signal transduction	SLA	T-cell receptor signaling	0.23
SLA	T-cell receptor signaling	TAGAP	T-cell activation	0.57
SLA	T-cell receptor signaling	CXCL13	Chemokine ligand	0.05
RHOH	T-cell antigen receptor signaling	TIGIT	T cell immunoreceptor	0.07
TIGIT	T cell immunoreceptor	APOBEC3H	Immune system process	-0.10
TRIM22	Interferon gamma signaling	SLFN5	Cell differentiation	-0.29
PPP1R16B	PI3K/AKT signaling pathway	GZMM	Immune system process	-0.23
LY9	Immunomodulatory receptors	CLECL1	Immune system process	0.46
CXCL13	Chemokine ligand	CD7	T-cell surface antigen	-0.18

### Validation of the PRSM in the GEO Samples

For the purpose of verifying the predictive effect of RS in different data-sets, we applied the PRSM to 156 COAD samples from GEO database (GSE103479) as a validation group. They were also classified into HRG and LRG (**Supplementary Table 2**), and survival curve was plotted. The result showed the consistency with the model group, because the OS of HRG was significantly lower than that of LRG (p=0.014) (**Figure 3E**, **Supplementary Table 2**). Hence, it was proved that the PRSM had accurate prediction value.

### Immune Infiltration Within Different Risk Groups

**Figure 4:**
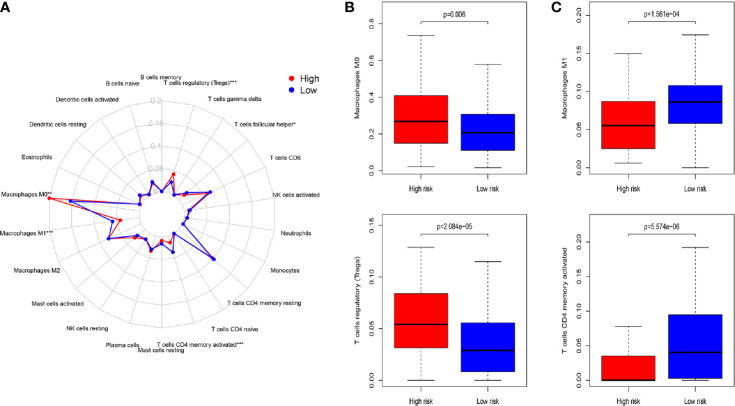
**(A)** Summary of the 22 immune cell types abundance estimated by “CIBERSORT” for different risk groups. **(B)** The differences of 22 immune cell types abundance within different risk groups. Treg cells (*p* < 0.001) and macrophage M0 (*p*=0.015) were significantly highly expressed in the high-risk group. **(C)** Activated memory CD4^+^ T cells and macrophage M1 were significantly higher in the low-risk group (all *p* < 0.001). *P*-values were based on t-test. (*P < 0.05, **P < 0.01, ***P < 0.001).

For the aim of exploring the specific cell types that caused differences in immune infiltration between the HRG and LRG, we used “CIBERSORT” to estimate the immune cell types abundance of the samples. The Wilcoxon rank sum test was performed to analyze the differences of 22 immune cell types abundance within HRG and LRG (**Figure 4A**). The results indicated that Treg cells (p<0.001) and macrophage M0 (p=0.015) were highly expressed in the HRG (**Figure 4B**). Activated memory CD4^+^ T cells and macrophage M1 were significantly highly expressed in the LRG (all p<0.001) (**Figure 4C**). The results showed the specific immune-related reasons for the poor prognosis in HRG.

### Functional Analysis and Identification of Key IRGs

**Figure 5:**
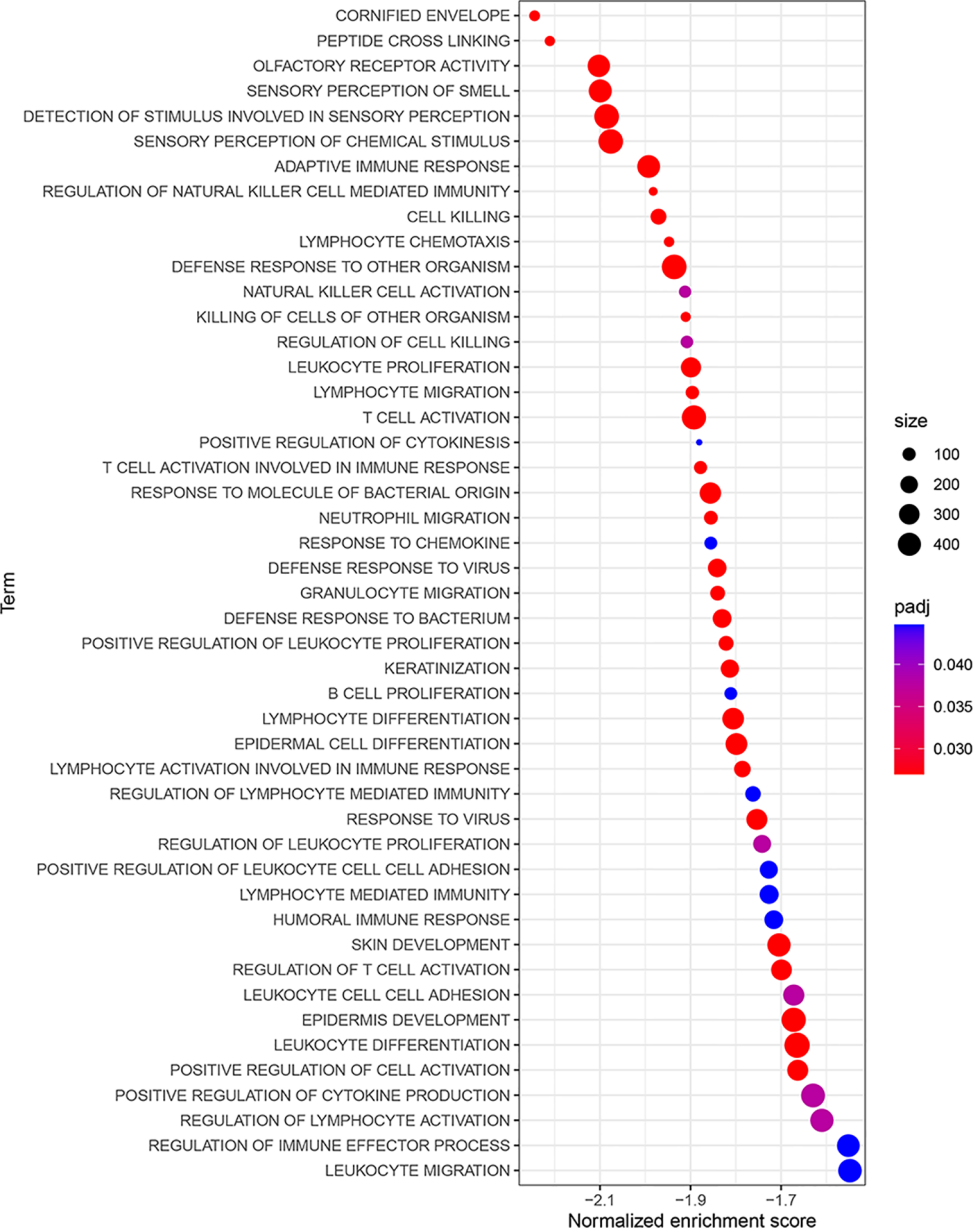
GO-related GSEA between different risk groups. The NES was regarded as the primary statistic for examining GSEA enrichment results. GO, gene ontology; GSEA, gene set enrichment analysis; NES, normalized enrichment score.

To study the significant changes of molecular function (MF), biological process (BP) and cellular component (CC), we conducted GO-related GSEA between HRG and LRG. The results showed that some terms were highly enriched in LRG, including lymphocyte chemotaxis, lymphocyte migration, T cell activation, positive regulation of cytokinesis, etc. (**Figure 5**, **Supplementary Table 3**). These enriched immune-related terms provided evidence for the molecular mechanism of PRSM.

**Figure 6:**
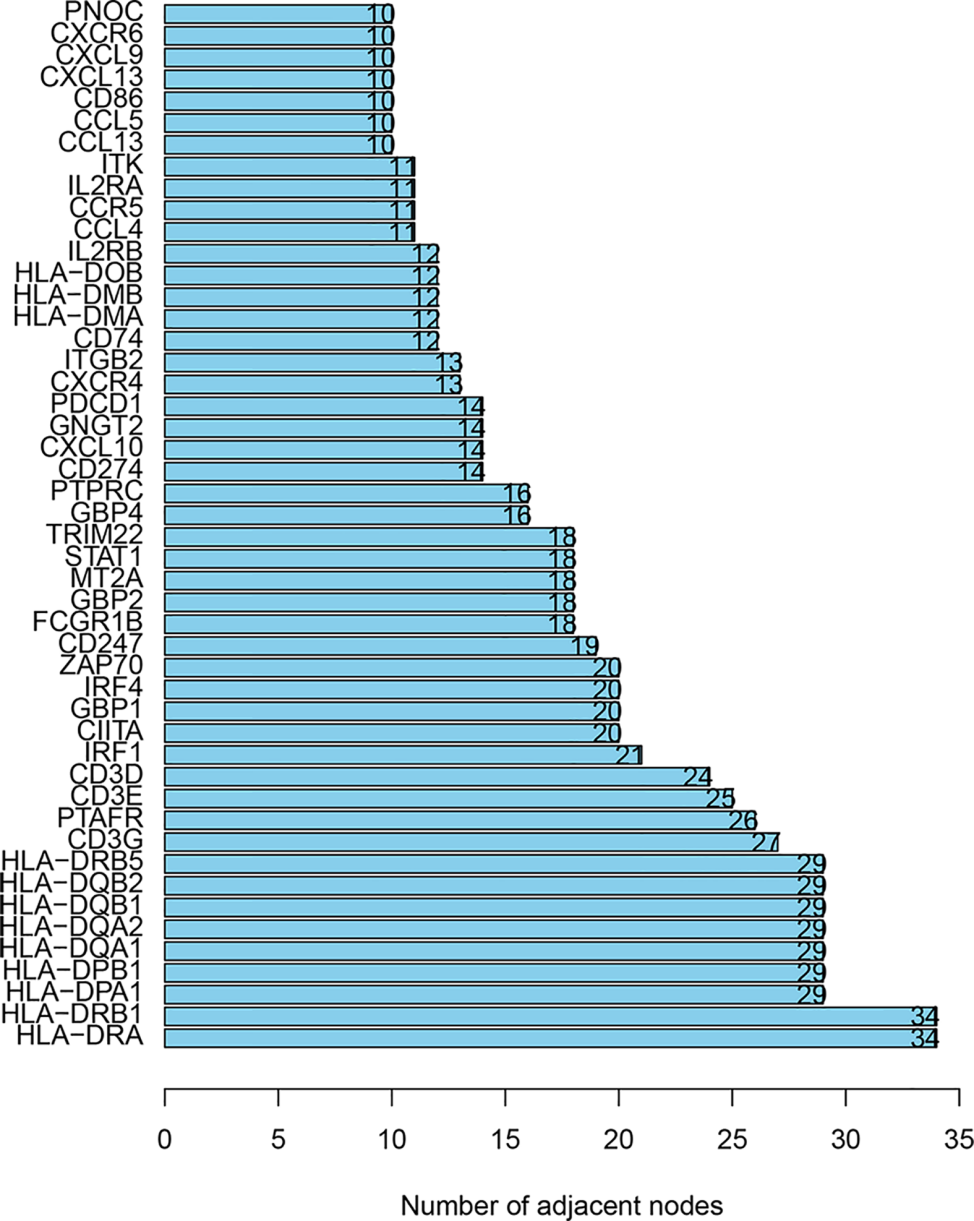
The genes with more than 10 interaction nodes in the PPI analyses. PPI, protein-protein interaction.

**Figure 7:**
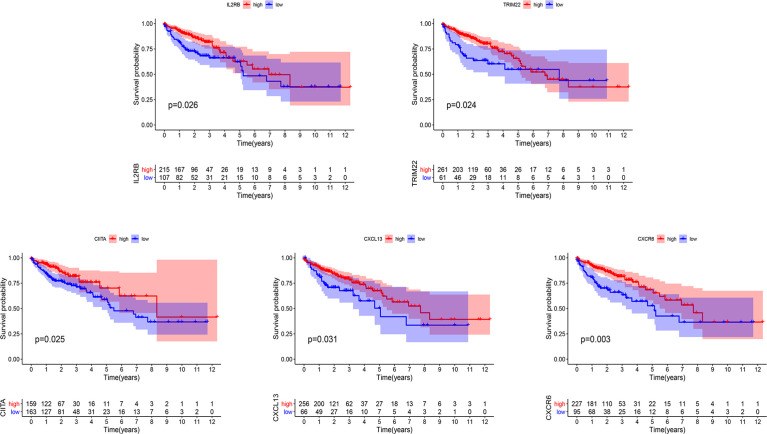
Kaplan-Meier curves of overall survival in five prognostic key IRGs. IRGs, immune related genes.

In order to determine the prognostic key IRGs in PRSM, we constructed a series of analyses of 215 relatively critical IRGs, including PPI, GO, and KEGG analyzes (**Supplementary Figure 1**). The genes with more than 10 interaction nodes in the network (**Figure 6**) were selected and intersected with IRGPs in PRSM. The genes intersection included IL2RB, TRIM22, CIITA, CXCL13, and CXCR6. Survival curves of these five genes showed that the prognosis of high expression group was better than that of low expression group (all p<0.05) (**Figure 7**). Also, we investigated the correlation between these five genes and clinical stages. The results indicated that the expression level of these five genes was higher in the earlier stage, the significant statistical differences were found between at least two clinical stages (**Supplementary Figure 2**).

**Figure 8:**
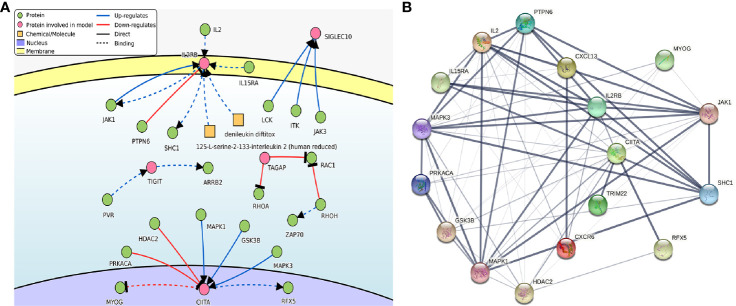
**(A)** The causal interaction of key gene analysis in DisNor. **(B)** The PPI analyses between key genes and directly interacted genes. The thickness of the solid line represents the strength of the relationship. PPI, protein-protein interaction.

DisNor revealed the first neighbor of disease-related genes in the database, in which we conducted the analysis of the genes in PRSM. The results included two key genes which were IL2RB and CIITA, and the genes as well as binding sites that interacted with them directly. IL2, JAK1, IL15RA, and PTPN6 lied upstream of IL2RB, JAK1, and SHC1 lied downstream. PRKACA, HDAC2, MAPK1, GSK3B, and MAPK3 lied upstream of CIITA, MYOG, and RFX5 lied downstream (**Figure 8A**). It was shown by the PPI analysis that there were complex and strong interactions between genes above and the other three key genes (**Figure 8B**). Particularly, IL2 interacted with five key genes, and JAK1 interacted with four key genes.

## Discussion

As early as 1990, Bufill proposed that proximal and distal CRC are two distinct tumors with obvious differences in epidemiology, pathology, cytogenetics, and molecular characteristics (3). It was also found by other studies that biomarkers for the prognosis of colon cancer, including microsatellite instability-high (23), CpG island methylator phenotype-high (24), RAS (25), phosphoinositide 3-kinase pathway (26), and BRAF mutations (24). Oncologists treated the patients individually based on these biomarkers in combination with tumor locations (27, 28). In addition, immune-related biomarkers can provide significant prognostic value as well as regulatory targets for immunotherapy (29). Therefore, we decided to explore the immunological differences between LCCs and RCCs, so as to figure out the IRGs that caused prognostic differences and provide a molecular basis for immunotherapy.

Risk models established by most studies used gene expression as a factor, which required appropriate standardization for unification. In the meantime, considering the inherent biological differences of different tumor samples and the errors caused by the sequencing platform, we chose a new method to construct the model factors. We only needed to compare the expression levels of two genes in the same sample by this method, making full use of the data while eliminating measurement errors in different samples. As shown in this study, we established IRGPs with application of a series of progressive analysis methods. Furthermore, we screened 16 prognostic-related IRGPs through PRSM, whose risk classification was evaluated and verified. In the analysis of immune cell types, we found that Treg cells and M0 macrophages had significantly high infiltration in the high-risk group. As one of the shapers of inhibitory TAIM, Treg cells produce immunosuppressive cytokines interleukin (IL)-2 and -10 to down-regulate the function of antigen-presenting cells (30). Treg cells also deprive co-stimulatory signal to responder T cells by down-modulating CD80/CD86 expression (31). In multiple cancers, Treg cells are associated with poor prognosis (32). Meanwhile, M0 macrophages may be related with the distant metastasis and prognosis of COAD (33). Macrophages M1 and activated memory CD4^+^ T cells were highly expressed in the low-risk group. As we all known, M1 macrophages, as recognized anti-tumor immune cells, have strong tumoricidal activity. It express proinflammatory cytokines to promote T-helper 1 response, and also produce reactive nitrogen and oxygen intermediates (34). Additionally, different studies have also proved that activated memory CD4^+^ T cells were the key instruments of tumor cure (35–37). CD4^+^ T cells can kill cancer cells directly, or kill tumor cells by stimulating and recruiting CD8^+^ T cells and other various immune cells indirectly (38). These evidences explain the prognostic differences caused by the different immune infiltration in the HRG and LRG.

In recent years, researchers have conducted in-depth studies on IRGs that lead to differences of immune infiltration in biliary tract cancers, and found that CTLA4 could affect chemotherapy resistance and prognosis through activation of Treg cells (39). Although researchers have evaluated prognostic IRGs in CRC, they have not independently analyzed the LCCs and RCCs with distinguishing characteristics (40–42). The key prognostic IRGs affecting the immune infiltration between LCCs and RCCs have not been explored yet. In our study, we screened five prognostic key IRGs from PRSM, in which IL2RB has relation with cytokines, TRIM22 and CIITA are transcription factors, CXCL13 and CXCR6 are related with chemokines. Interleukin 2 receptor (IL2R) participates in the immune response mediated by T cells. The binding of IL-2 and IL2R activate both NK and T cells potentially, which has a killing efficacy on tumors (43). It is confirmed that the TRIM22 played many crucial roles in different biological processes, from inflammatory to tumorigenesis. In endometrial cancer, TRIM22 is proven to inhibit tumor growth by NF-κB signaling pathway, and conferred a favorable prognosis (44). CIITA is the regulator of the major histocompatibility complex gene expression (45). CIITA promotes T lymphocyte activation and adaptive immunity by regulating MHCII transcription (46). In colorectal and gastric cancer, the reactivation of CIITA activates the immune system and contributes to the anti-tumor immune response (47). Also in lung adenocarcinoma, enforced expression of CIITA increases T cell infiltration and sensitivity to anti-PD-1 therapy (48). In non-small cell lung cancer (NSCLC), as the ligand of CXCR5, CXCL13 has been reported to be highly expressed in CD8^+^ lymphocyte populations with high PD-1 expression, which can attract other immune cells to TAIM and predict response to anti-PD-1 therapy strongly (49). As the receptor of CXCL16, CXCR6 has a controversial effect on tumors. In NSCLC and prostate cancer, the increase of CXCL16 and CXCR6 is related with the poor prognostic features of patients (50–52). In colorectal cancer, CXCL16 secreted by cancer cells recruits CD4^+^ and CD8^+^ T cells (53, 54). Irradiation induces the expression of CXCL16 in breast cancer cells, enhancing the migration of NK cells with high CXCR6 expression to kill tumor cells (55). In other cancers such as melanoma, lung adenocarcinoma and glioma, similar prognostic IRG signatures have also been explored and identified. Although IRGs are not exactly the same, they were all mainly enriched in pathways closely associated microenvironment, and affected the abundance of CD4^+^, CD8^+^ T cells, and macrophages (56–58). The above researches have provided evidence for the prognostic key IRGs whose potential as a regulatory target for immunotherapy is implied as well.

Limitations are as following: firstly, the establishment of PRSM is based on gene expression. The high price of RNA sequencing technology is not suitable for clinical promotion. Given this, we screened out a few prognostic key IRGs in the subsequent analyses. However, we require additional experiments to investigate the specific function of these prognostic key IRGs. Secondly, other than the model group platform, only one gene set was selected as the verification group, and more independent real-world cohorts are required for validation to ensure the accuracy and robustness of the model.

In conclusion, we constructed PRSM based on the differentially expressed IRGs of LCCs and RCCs. While applying PRSM to provide prognostic value, we gained a deeper insight in immune-related mechanisms. Meanwhile we predicted and identified five prognostic key IRGs, hoping to provide some basis for identifying the benefits of immunotherapy and immunomodulatory.

## Data Availability Statement

Publicly available datasets were analyzed in this study. These data can be found here: TCGA database (http://cancergenome.nih.gov/) and the NCBI Gene Expression Omnibus (GSE103479) (https://www.ncbi.nlm.nih.gov/).

## Author Contributions

B-BC and Y-LL designed the study. J-NG, M-QL, and S-HD drafted the manuscript. J-NG, M-QL, and S-HD collected, analyzed, and interpreted the data. J-NG, CC, and YN participated in revising the manuscript. All authors contributed to the article and approved the submitted version.

## Funding

This work was supported by Natural Science Foundation of Heilongjiang Province of China (ZD2017019), Nn10 Program of Harbin Medical University Cancer Hospital (Nn102017-02), the Post-doctoral Scientific Research Developmental Fund of Heilongjiang (LBH-Q18085), and Harbin Medical University Cancer Hospital Preeminence Youth Fund (JCQN2019-04).

## Conflict of Interest

The authors declare that the research was conducted in the absence of any commercial or financial relationships that could be construed as a potential conflict of interest.

## Supplementary Material

Supplementary Figure 1 Gene ontology (GO) and Kyoto Encyclopedia of Genes and Genomes (KEGG) analyzes in 215 relatively critical IRGs. IRGs, immune related genes.

Supplementary Figure 2 The correlation between 5 prognostic key IRGs and clinical stages.
