# Supplementary figures and images for: Prognostic Immune-Related Analysis Based on Differentially Expressed Genes in Left- and Right-Sided Colon Adenocarcinoma
Source: Front Oncol. 2021 Mar 8;11:640196. doi: 10.3389/fonc.2021.640196 (PMC7982460; doi:10.3389/fonc.2021.640196)

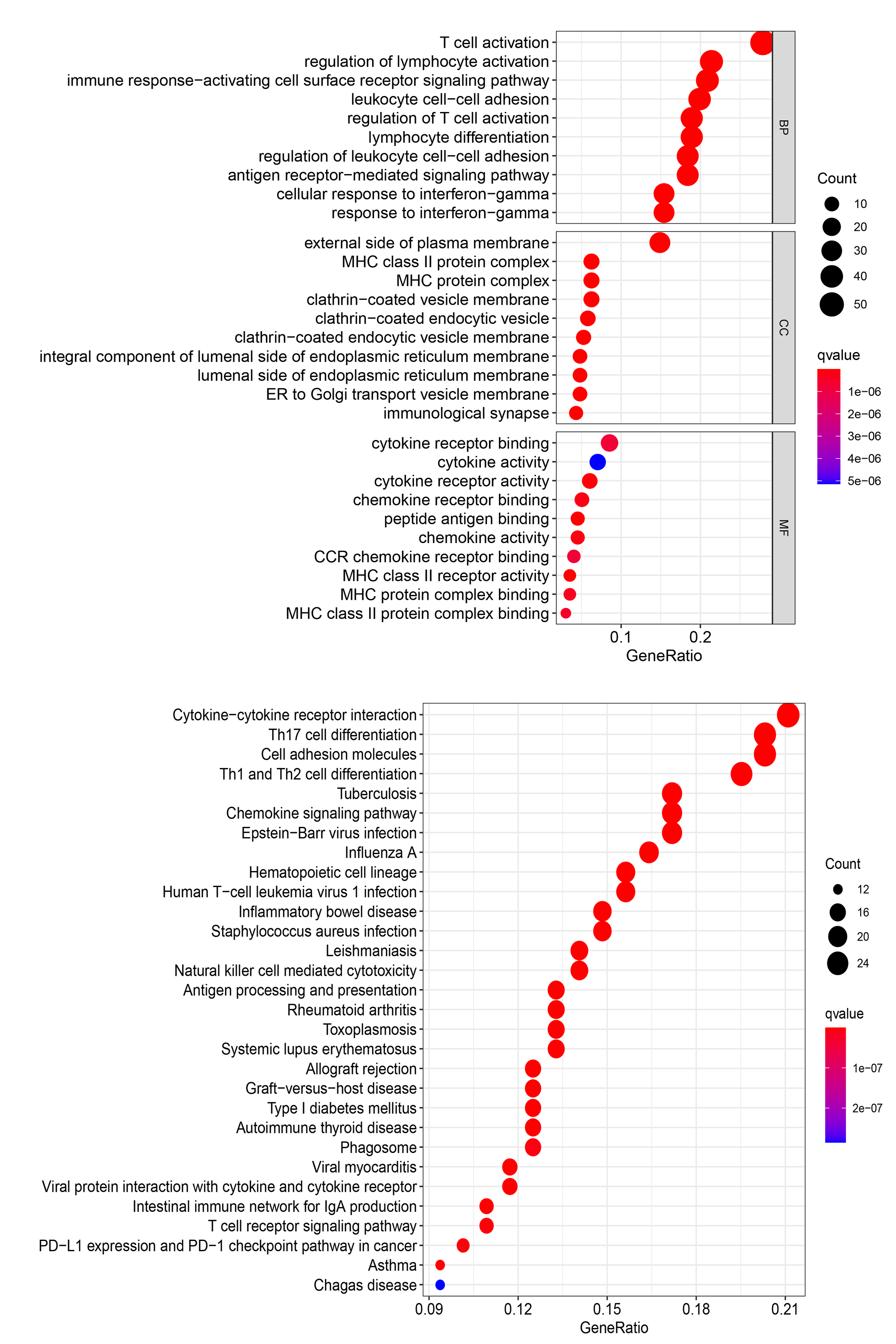

Supplement: Supplementary Figure 1 — Gene ontology (GO) and Kyoto Encyclopedia of Genes and Genomes (KEGG) analyzes in 215 relatively critical IRGs. IRGs, immune related genes. [file Image_1.tif]

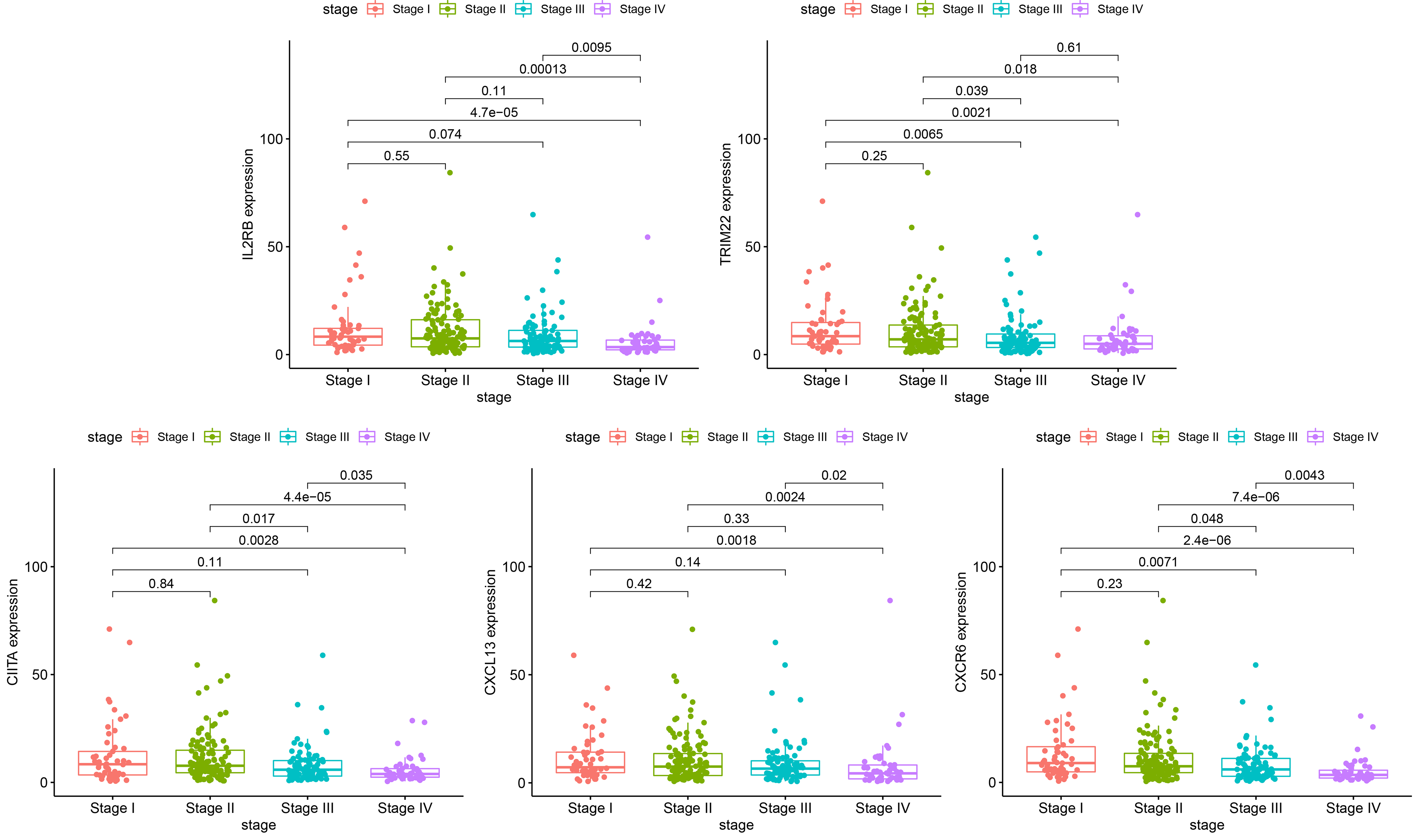

Supplement: Supplementary Figure 2 — The correlation between 5 prognostic key IRGs and clinical stages. [file Image_2.tif]
