# Supplementary material for: Prognostic Immune-Related Analysis Based on Differentially Expressed Genes in Left- and Right-Sided Colon Adenocarcinoma
Source: Front Oncol. 2021 Mar 8;11:640196. doi: 10.3389/fonc.2021.640196 (PMC7982460; doi:10.3389/fonc.2021.640196)
Supplement: Supplementary file 3 [file Table_1.docx]

Table.S1.A total of 69 IRGPs were selected by univariate-Cox proportional hazards regression.

| Gene pairs | HR | HR.95L | HR.95H | Cox *p-*value |
| --- | --- | --- | --- | --- |
| IL2RA\|SIGLEC1 | 0.55 | 0.35 | 0.87 | 0.012 |
| NCKAP1L\|PPP1R16B | 2.03 | 1.07 | 3.84 | 0.030 |
| NCKAP1L\|CXCL13 | 1.71 | 1.08 | 2.69 | 0.021 |
| CD300A\|SASH3 | 1.86 | 1.16 | 2.99 | 0.010 |
| SCIMP\|CTLA4 | 1.75 | 1.08 | 2.83 | 0.022 |
| CD300LF\|CCR5 | 1.69 | 1.07 | 2.66 | 0.024 |
| SASH3\|GIMAP4 | 0.45 | 0.22 | 0.94 | 0.032 |
| LSP1\|CXCL9 | 1.91 | 1.21 | 3.00 | 0.005 |
| APBB1IP\|SLA | 1.62 | 1.03 | 2.54 | 0.037 |
| APBB1IP\|TAGAP | 2.51 | 1.38 | 4.57 | 0.002 |
| APBB1IP\|CXCL13 | 1.71 | 1.08 | 2.71 | 0.023 |
| SIGLEC10\|TAGAP | 2.59 | 1.37 | 4.91 | 0.003 |
| SIGLEC10\|KLRB1 | 1.60 | 1.01 | 2.53 | 0.046 |
| CD2\|CD163 | 0.53 | 0.33 | 0.83 | 0.006 |
| NFAM1\|SLA | 1.70 | 1.08 | 2.69 | 0.022 |
| NFAM1\|TBC1D10C | 1.73 | 1.05 | 2.84 | 0.030 |
| SLAMF7\|HAPLN3 | 0.48 | 0.24 | 0.97 | 0.040 |
| SLAMF7\|NKG7 | 0.37 | 0.15 | 0.91 | 0.031 |
| SLAMF7\|CD163 | 0.60 | 0.36 | 1.00 | 0.049 |
| CCR5\|ARHGAP25 | 0.63 | 0.40 | 1.00 | 0.048 |
| CCR5\|SLC2A5 | 0.59 | 0.36 | 0.95 | 0.031 |
| CCR5\|IKZF3 | 0.54 | 0.33 | 0.89 | 0.016 |
| FAM78A\|SIRPG | 1.78 | 1.05 | 3.02 | 0.033 |
| FAM78A\|CIITA | 1.82 | 1.14 | 2.89 | 0.011 |
| IL2RB\|ODF3B | 0.47 | 0.24 | 0.91 | 0.025 |
| DOCK2\|CXCR6 | 1.64 | 1.01 | 2.68 | 0.047 |
| ARHGAP25\|TAGAP | 2.38 | 1.39 | 4.09 | 0.002 |
| ARHGAP25\|PPP1R16B | 2.36 | 1.40 | 3.96 | 0.001 |
| ARHGAP25\|CXCL13 | 1.61 | 1.02 | 2.54 | 0.039 |
| FCGR3A\|CXCL10 | 1.67 | 1.02 | 2.72 | 0.042 |
| SLA\|TAGAP | 1.86 | 1.02 | 3.38 | 0.042 |
| SLA\|EVL | 0.45 | 0.23 | 0.88 | 0.020 |
| SLA\|CXCL13 | 1.78 | 1.13 | 2.79 | 0.013 |
| TAGAP\|EVL | 0.46 | 0.21 | 1.00 | 0.049 |
| TAGAP\|NFATC1 | 0.62 | 0.39 | 0.99 | 0.045 |
| CXCR6\|RASAL3 | 0.44 | 0.25 | 0.79 | 0.006 |
| CXCR6\|PIK3CD | 0.58 | 0.35 | 0.97 | 0.036 |
| SIRPG\|ZAP70 | 0.62 | 0.39 | 0.99 | 0.046 |
| SIGLEC1\|P2RY10 | 1.93 | 1.10 | 3.41 | 0.023 |
| SIGLEC1\|CTLA4 | 1.95 | 1.24 | 3.07 | 0.004 |
| SIGLEC1\|RASAL3 | 2.21 | 1.33 | 3.68 | 0.002 |
| TIGIT\|CD72 | 0.50 | 0.29 | 0.89 | 0.017 |
| TIGIT\|GZMM | 0.35 | 0.18 | 0.68 | 0.002 |
| TIGIT\|NFATC1 | 0.45 | 0.27 | 0.77 | 0.003 |
| P2RY10\|RASGRP1 | 0.62 | 0.38 | 0.99 | 0.044 |
| CTLA4\|HIVEP3 | 0.59 | 0.37 | 0.96 | 0.032 |
| PTPRC\|EVL | 0.62 | 0.39 | 0.98 | 0.042 |
| TBC1D10C\|CXCL13 | 1.90 | 1.20 | 3.01 | 0.006 |
| TBC1D10C\|CIITA | 1.72 | 1.09 | 2.72 | 0.019 |
| FCGR1B\|CLECL1 | 1.67 | 1.06 | 2.63 | 0.026 |
| HAPLN3\|SAMD9L | 2.26 | 1.24 | 4.12 | 0.008 |
| PPP1R16B\|GZMM | 0.62 | 0.39 | 0.97 | 0.036 |
| PPP1R16B\|IKZF3 | 0.60 | 0.38 | 0.94 | 0.027 |
| PPP1R16B\|NFATC1 | 0.50 | 0.31 | 0.79 | 0.003 |
| CD8A\|CXCL13 | 1.59 | 1.00 | 2.50 | 0.048 |
| ITK\|NFATC1 | 0.46 | 0.21 | 0.99 | 0.047 |
| GZMM\|CXCL13 | 1.87 | 1.16 | 3.03 | 0.011 |
| LY9\|CLECL1 | 1.62 | 1.02 | 2.55 | 0.039 |
| CXCL13\|CD7 | 0.59 | 0.37 | 0.94 | 0.026 |
| CXCL13\|ODF3B | 0.56 | 0.33 | 0.95 | 0.030 |
| CXCL10\|IFI16 | 0.63 | 0.40 | 1.00 | 0.048 |
| CXCL10\|IFIT3 | 0.57 | 0.36 | 0.90 | 0.017 |
| CD27\|ODF3B | 0.47 | 0.23 | 0.98 | 0.044 |
| TRAT1\|TESPA1 | 1.67 | 1.05 | 2.63 | 0.029 |
| TRAT1\|CLECL1 | 1.96 | 1.14 | 3.37 | 0.015 |
| TCAF2\|VNN2 | 1.86 | 1.19 | 2.93 | 0.007 |
| TRIM69\|FAS | 1.95 | 1.24 | 3.08 | 0.004 |
| MMP12\|TYMP | 0.61 | 0.39 | 0.96 | 0.032 |
| JAK2\|FAS | 1.62 | 1.02 | 2.59 | 0.042 |

Table.S2.Risk grouping of patients in model and validation groups.

| ID | Cohort | Risk Score | Risk groups |
| --- | --- | --- | --- |
| TCGA-AD-6965 | TCGA | 0.05 | low |
| TCGA-CM-5862 | TCGA | 1.27 | high |
| TCGA-AZ-4308 | TCGA | 0.94 | low |
| TCGA-G4-6298 | TCGA | 1.70 | high |
| TCGA-AZ-6601 | TCGA | 1.24 | high |
| TCGA-A6-6138 | TCGA | -0.45 | low |
| TCGA-AY-A8YK | TCGA | 1.10 | high |
| TCGA-D5-5538 | TCGA | 1.09 | high |
| TCGA-AA-A00D | TCGA | 0.46 | low |
| TCGA-DM-A288 | TCGA | 1.75 | high |
| TCGA-NH-A50T | TCGA | 0.92 | low |
| TCGA-D5-5539 | TCGA | 1.48 | high |
| TCGA-AA-3867 | TCGA | 0.85 | low |
| TCGA-DM-A1DB | TCGA | 1.05 | high |
| TCGA-CM-5341 | TCGA | 0.50 | low |
| TCGA-G4-6299 | TCGA | 1.25 | high |
| TCGA-G4-6306 | TCGA | 0.92 | low |
| TCGA-AA-A01V | TCGA | 1.12 | high |
| TCGA-AU-3779 | TCGA | 1.41 | high |
| TCGA-A6-2679 | TCGA | 1.34 | high |
| TCGA-F4-6855 | TCGA | 1.72 | high |
| TCGA-DM-A28F | TCGA | 1.74 | high |
| TCGA-DM-A28G | TCGA | 0.96 | low |
| TCGA-AA-A01P | TCGA | 1.06 | high |
| TCGA-AA-3860 | TCGA | -0.27 | low |
| TCGA-3L-AA1B | TCGA | 0.56 | low |
| TCGA-AD-5900 | TCGA | 0.01 | low |
| TCGA-G4-6322 | TCGA | 1.18 | high |
| TCGA-CK-6746 | TCGA | 0.52 | low |
| TCGA-F4-6703 | TCGA | 0.16 | low |
| TCGA-D5-6541 | TCGA | 0.23 | low |
| TCGA-AA-3972 | TCGA | 0.68 | low |
| TCGA-CM-4743 | TCGA | 0.82 | low |
| TCGA-F4-6459 | TCGA | 1.49 | high |
| TCGA-A6-A566 | TCGA | 1.12 | high |
| TCGA-G4-6302 | TCGA | 0.79 | low |
| TCGA-G4-6625 | TCGA | -0.65 | low |
| TCGA-AA-A01T | TCGA | 1.92 | high |
| TCGA-CM-5863 | TCGA | 0.78 | low |
| TCGA-D5-7000 | TCGA | -0.82 | low |
| TCGA-CK-5914 | TCGA | -0.23 | low |
| TCGA-AZ-6600 | TCGA | 1.48 | high |
| TCGA-G4-6627 | TCGA | -0.10 | low |
| TCGA-NH-A50V | TCGA | 0.83 | low |
| TCGA-AA-A01Q | TCGA | 0.78 | low |
| TCGA-CM-6164 | TCGA | 0.31 | low |
| TCGA-NH-A8F8 | TCGA | 0.61 | low |
| TCGA-AA-3522 | TCGA | 0.47 | low |
| TCGA-AY-A71X | TCGA | 1.58 | high |
| TCGA-CM-4746 | TCGA | 0.63 | low |
| TCGA-AA-3848 | TCGA | 1.78 | high |
| TCGA-A6-6142 | TCGA | 1.29 | high |
| TCGA-AA-3495 | TCGA | 0.10 | low |
| TCGA-QL-A97D | TCGA | -0.23 | low |
| TCGA-AZ-4615 | TCGA | 0.13 | low |
| TCGA-D5-6927 | TCGA | -0.56 | low |
| TCGA-DM-A28M | TCGA | 1.23 | high |
| TCGA-DM-A1D4 | TCGA | 1.09 | high |
| TCGA-D5-6920 | TCGA | 0.21 | low |
| TCGA-AA-3861 | TCGA | 1.01 | high |
| TCGA-AA-A01S | TCGA | 1.97 | high |
| TCGA-AA-A022 | TCGA | 1.47 | high |
| TCGA-F4-6806 | TCGA | 0.10 | low |
| TCGA-AZ-5403 | TCGA | 0.77 | low |
| TCGA-D5-6930 | TCGA | 0.00 | low |
| TCGA-CM-6677 | TCGA | -0.23 | low |
| TCGA-AA-A00W | TCGA | 0.48 | low |
| TCGA-5M-AAT6 | TCGA | 0.01 | low |
| TCGA-CM-6675 | TCGA | -0.05 | low |
| TCGA-G4-6323 | TCGA | -0.41 | low |
| TCGA-AA-3675 | TCGA | 0.30 | low |
| TCGA-CM-6172 | TCGA | 0.95 | low |
| TCGA-AZ-4313 | TCGA | 0.30 | low |
| TCGA-D5-6540 | TCGA | 0.41 | low |
| TCGA-AD-A5EK | TCGA | 0.59 | low |
| TCGA-AA-3518 | TCGA | -0.14 | low |
| TCGA-AA-A01F | TCGA | 1.05 | high |
| TCGA-AA-A01G | TCGA | 0.65 | low |
| TCGA-AA-3526 | TCGA | -0.49 | low |
| TCGA-AA-3560 | TCGA | -0.05 | low |
| TCGA-AA-A02W | TCGA | 0.97 | low |
| TCGA-AZ-6607 | TCGA | 1.81 | high |
| TCGA-AZ-6605 | TCGA | 0.46 | low |
| TCGA-G4-6303 | TCGA | 1.19 | high |
| TCGA-AD-A5EJ | TCGA | 0.77 | low |
| TCGA-AA-3534 | TCGA | 0.07 | low |
| TCGA-AA-3956 | TCGA | 0.26 | low |
| TCGA-DM-A28H | TCGA | 0.96 | low |
| TCGA-DM-A1HA | TCGA | 1.51 | high |
| TCGA-A6-2671 | TCGA | 2.02 | high |
| TCGA-T9-A92H | TCGA | -0.21 | low |
| TCGA-AA-A017 | TCGA | 0.94 | low |
| TCGA-CM-6162 | TCGA | 0.03 | low |
| TCGA-A6-6648 | TCGA | 0.76 | low |
| TCGA-G4-6628 | TCGA | 0.09 | low |
| TCGA-CA-6716 | TCGA | 1.11 | high |
| TCGA-AA-3524 | TCGA | -0.05 | low |
| TCGA-AY-6386 | TCGA | 0.48 | low |
| TCGA-5M-AAT4 | TCGA | 1.97 | high |
| TCGA-AA-A00U | TCGA | 0.63 | low |
| TCGA-A6-6649 | TCGA | 0.63 | low |
| TCGA-A6-5656 | TCGA | 0.26 | low |
| TCGA-D5-6928 | TCGA | -0.56 | low |
| TCGA-AA-3845 | TCGA | 1.35 | high |
| TCGA-DM-A1D6 | TCGA | 1.17 | high |
| TCGA-CM-4752 | TCGA | 0.30 | low |
| TCGA-CK-5912 | TCGA | 0.60 | low |
| TCGA-A6-6650 | TCGA | -0.07 | low |
| TCGA-NH-A8F7 | TCGA | 1.17 | high |
| TCGA-AA-3678 | TCGA | 0.66 | low |
| TCGA-CM-5348 | TCGA | 1.18 | high |
| TCGA-CM-6678 | TCGA | 0.67 | low |
| TCGA-CM-4744 | TCGA | 0.00 | low |
| TCGA-AD-6888 | TCGA | 0.94 | low |
| TCGA-AA-3952 | TCGA | 2.57 | high |
| TCGA-F4-6704 | TCGA | 1.53 | high |
| TCGA-CM-6166 | TCGA | 1.56 | high |
| TCGA-AA-A004 | TCGA | 1.56 | high |
| TCGA-WS-AB45 | TCGA | 0.78 | low |
| TCGA-DM-A28K | TCGA | 1.61 | high |
| TCGA-NH-A6GA | TCGA | 1.34 | high |
| TCGA-DM-A0XD | TCGA | 0.97 | high |
| TCGA-F4-6460 | TCGA | 1.00 | high |
| TCGA-AZ-6608 | TCGA | 1.48 | high |
| TCGA-G4-6311 | TCGA | 1.61 | high |
| TCGA-AA-A024 | TCGA | 1.23 | high |
| TCGA-CM-5349 | TCGA | 0.73 | low |
| TCGA-CM-6679 | TCGA | 0.54 | low |
| TCGA-AD-6889 | TCGA | 0.60 | low |
| TCGA-CK-5916 | TCGA | 0.10 | low |
| TCGA-A6-A56B | TCGA | 1.34 | high |
| TCGA-AA-A00O | TCGA | 0.91 | low |
| TCGA-G4-6315 | TCGA | 1.02 | high |
| TCGA-AA-3869 | TCGA | 1.11 | high |
| TCGA-A6-6137 | TCGA | -0.44 | low |
| TCGA-G4-6317 | TCGA | 1.51 | high |
| TCGA-G4-6295 | TCGA | 0.03 | low |
| TCGA-CM-6163 | TCGA | 0.54 | low |
| TCGA-A6-6782 | TCGA | 1.01 | high |
| TCGA-CK-4952 | TCGA | 1.53 | high |
| TCGA-G4-6297 | TCGA | 0.86 | low |
| TCGA-AA-3870 | TCGA | 1.41 | high |
| TCGA-A6-5662 | TCGA | 1.47 | high |
| TCGA-D5-6532 | TCGA | 1.40 | high |
| TCGA-AA-3715 | TCGA | 0.65 | low |
| TCGA-CM-5864 | TCGA | 0.27 | low |
| TCGA-DM-A28A | TCGA | 1.48 | high |
| TCGA-DM-A285 | TCGA | 1.90 | high |
| TCGA-SS-A7HO | TCGA | 1.61 | high |
| TCGA-CK-5913 | TCGA | -0.03 | low |
| TCGA-AA-A02Y | TCGA | 0.41 | low |
| TCGA-5M-AATE | TCGA | 0.67 | low |
| TCGA-AA-A00Z | TCGA | 1.87 | high |
| TCGA-AA-3710 | TCGA | 0.21 | low |
| TCGA-AY-6197 | TCGA | -0.05 | low |
| TCGA-DM-A280 | TCGA | 2.11 | high |
| TCGA-AA-3930 | TCGA | 1.00 | high |
| TCGA-AA-A00E | TCGA | 1.01 | high |
| TCGA-AA-A01I | TCGA | 0.69 | low |
| TCGA-AA-3815 | TCGA | 0.53 | low |
| TCGA-AA-3875 | TCGA | 0.64 | low |
| TCGA-QG-A5YW | TCGA | -0.06 | low |
| TCGA-AA-A00R | TCGA | 0.37 | low |
| TCGA-AA-A01X | TCGA | 1.47 | high |
| TCGA-A6-2683 | TCGA | 1.94 | high |
| TCGA-G4-6588 | TCGA | 1.48 | high |
| TCGA-AA-3655 | TCGA | 0.66 | low |
| TCGA-AA-3684 | TCGA | 2.25 | high |
| TCGA-AA-A02E | TCGA | 2.20 | high |
| TCGA-A6-2675 | TCGA | 0.68 | low |
| TCGA-AA-A01K | TCGA | 0.58 | low |
| TCGA-AA-A02R | TCGA | 0.80 | low |
| TCGA-A6-6780 | TCGA | -0.16 | low |
| TCGA-CK-4950 | TCGA | -0.39 | low |
| TCGA-AA-A00J | TCGA | 1.25 | high |
| TCGA-F4-6854 | TCGA | 0.66 | low |
| TCGA-CM-6161 | TCGA | 0.24 | low |
| TCGA-AA-3554 | TCGA | 1.04 | high |
| TCGA-AA-A01C | TCGA | 0.90 | low |
| TCGA-AZ-6599 | TCGA | 1.76 | high |
| TCGA-CA-5796 | TCGA | 1.23 | high |
| TCGA-CK-4948 | TCGA | 0.51 | low |
| TCGA-AA-A02J | TCGA | 0.98 | high |
| TCGA-AZ-6598 | TCGA | 1.25 | high |
| TCGA-G4-6309 | TCGA | 0.51 | low |
| TCGA-AA-3812 | TCGA | 2.34 | high |
| TCGA-CM-4751 | TCGA | 0.46 | low |
| TCGA-D5-6530 | TCGA | 0.03 | low |
| TCGA-AA-A03J | TCGA | 1.00 | high |
| TCGA-AY-4071 | TCGA | 1.76 | high |
| TCGA-AA-3979 | TCGA | 1.23 | high |
| TCGA-A6-5665 | TCGA | 1.08 | high |
| TCGA-AA-3663 | TCGA | 0.76 | low |
| TCGA-A6-5667 | TCGA | 0.78 | low |
| TCGA-AA-3712 | TCGA | 0.09 | low |
| TCGA-DM-A282 | TCGA | 1.51 | high |
| TCGA-AD-6964 | TCGA | 1.51 | high |
| TCGA-CK-6748 | TCGA | 0.73 | low |
| TCGA-NH-A6GC | TCGA | 1.40 | high |
| TCGA-AY-6196 | TCGA | 0.11 | low |
| TCGA-AA-3542 | TCGA | 1.46 | high |
| TCGA-AA-3864 | TCGA | -0.24 | low |
| TCGA-AD-6548 | TCGA | -0.45 | low |
| TCGA-4T-AA8H | TCGA | 0.71 | low |
| TCGA-CM-5868 | TCGA | 0.64 | low |
| TCGA-AU-6004 | TCGA | 0.20 | low |
| TCGA-AA-A029 | TCGA | 0.63 | low |
| TCGA-AA-A00Q | TCGA | 1.14 | high |
| TCGA-CM-5344 | TCGA | 1.12 | high |
| TCGA-CM-6674 | TCGA | 0.13 | low |
| TCGA-AA-3846 | TCGA | -0.34 | low |
| TCGA-DM-A1DA | TCGA | 1.76 | high |
| TCGA-DM-A1D7 | TCGA | 0.53 | low |
| TCGA-CK-4951 | TCGA | 0.90 | low |
| TCGA-D5-6531 | TCGA | 0.98 | high |
| TCGA-A6-5661 | TCGA | -0.73 | low |
| TCGA-AA-3984 | TCGA | -0.25 | low |
| TCGA-AY-4070 | TCGA | 0.54 | low |
| TCGA-A6-6652 | TCGA | 1.38 | high |
| TCGA-CA-6717 | TCGA | 0.27 | low |
| TCGA-DM-A28C | TCGA | 0.72 | low |
| TCGA-4N-A93T | TCGA | 1.24 | high |
| TCGA-CA-6715 | TCGA | 0.59 | low |
| TCGA-AA-3509 | TCGA | 0.52 | low |
| TCGA-AA-A03F | TCGA | 1.89 | high |
| TCGA-AA-3949 | TCGA | -0.95 | low |
| TCGA-AA-3538 | TCGA | 0.77 | low |
| TCGA-F4-6809 | TCGA | 0.75 | low |
| TCGA-QG-A5YX | TCGA | 0.48 | low |
| TCGA-RU-A8FL | TCGA | 0.76 | low |
| TCGA-AA-A02F | TCGA | 1.37 | high |
| TCGA-AA-3950 | TCGA | 0.77 | low |
| TCGA-CK-5915 | TCGA | 1.65 | high |
| TCGA-AZ-4323 | TCGA | 1.01 | high |
| TCGA-D5-6924 | TCGA | 0.35 | low |
| TCGA-DM-A1D0 | TCGA | 0.83 | low |
| TCGA-CK-6751 | TCGA | -0.28 | low |
| TCGA-AY-A54L | TCGA | 0.43 | low |
| TCGA-AA-3529 | TCGA | 1.40 | high |
| TCGA-AA-3543 | TCGA | 0.99 | high |
| TCGA-AZ-4614 | TCGA | 0.82 | low |
| TCGA-A6-2680 | TCGA | 1.75 | high |
| TCGA-AA-A00F | TCGA | 1.62 | high |
| TCGA-CM-6165 | TCGA | 0.23 | low |
| TCGA-F4-6808 | TCGA | 1.16 | high |
| TCGA-CM-6167 | TCGA | 0.55 | low |
| TCGA-AA-A00A | TCGA | 0.89 | low |
| TCGA-CM-5861 | TCGA | 1.34 | high |
| TCGA-AA-3821 | TCGA | 0.31 | low |
| TCGA-DM-A1D8 | TCGA | 1.43 | high |
| TCGA-AD-6901 | TCGA | 1.62 | high |
| TCGA-CA-6719 | TCGA | 0.84 | low |
| TCGA-AA-3496 | TCGA | 0.66 | low |
| TCGA-D5-6926 | TCGA | 0.73 | low |
| TCGA-AY-5543 | TCGA | -0.80 | low |
| TCGA-AZ-4616 | TCGA | 0.46 | low |
| TCGA-CM-4747 | TCGA | 1.68 | high |
| TCGA-G4-6626 | TCGA | 0.82 | low |
| TCGA-A6-6140 | TCGA | 0.08 | low |
| TCGA-D5-5540 | TCGA | -0.06 | low |
| TCGA-QG-A5YV | TCGA | 0.28 | low |
| TCGA-A6-3807 | TCGA | 0.20 | low |
| TCGA-AA-3520 | TCGA | 0.97 | high |
| TCGA-DM-A1D9 | TCGA | 0.78 | low |
| TCGA-AD-6890 | TCGA | 0.22 | low |
| TCGA-AA-3527 | TCGA | 1.38 | high |
| TCGA-AA-3693 | TCGA | 0.59 | low |
| TCGA-QG-A5Z2 | TCGA | -0.21 | low |
| TCGA-AA-3525 | TCGA | 0.78 | low |
| TCGA-AD-6895 | TCGA | -0.23 | low |
| TCGA-AA-3681 | TCGA | 0.51 | low |
| TCGA-D5-6922 | TCGA | 0.56 | low |
| TCGA-AA-3492 | TCGA | 1.46 | high |
| TCGA-AZ-6606 | TCGA | 1.02 | high |
| TCGA-CM-6169 | TCGA | 0.06 | low |
| TCGA-G4-6321 | TCGA | 0.40 | low |
| TCGA-AD-6963 | TCGA | 0.23 | low |
| TCGA-CM-6168 | TCGA | 0.33 | low |
| TCGA-CM-6170 | TCGA | 1.47 | high |
| TCGA-CM-6676 | TCGA | 0.76 | low |
| TCGA-AA-3664 | TCGA | -0.12 | low |
| TCGA-DM-A28E | TCGA | 0.29 | low |
| TCGA-AZ-4315 | TCGA | 0.33 | low |
| TCGA-AA-3696 | TCGA | 0.94 | low |
| TCGA-NH-A50U | TCGA | 1.78 | high |
| TCGA-CM-5860 | TCGA | 0.46 | low |
| TCGA-G4-6307 | TCGA | 1.21 | high |
| TCGA-AA-3977 | TCGA | -0.20 | low |
| TCGA-AM-5821 | TCGA | 0.54 | low |
| TCGA-AZ-6603 | TCGA | 0.86 | low |
| TCGA-AA-A02H | TCGA | 1.95 | high |
| TCGA-AZ-5407 | TCGA | 1.46 | high |
| TCGA-AA-A01Z | TCGA | 2.07 | high |
| TCGA-AA-3552 | TCGA | 1.30 | high |
| TCGA-D5-5541 | TCGA | 0.23 | low |
| TCGA-CK-4947 | TCGA | -0.93 | low |
| TCGA-DM-A0XF | TCGA | 0.93 | low |
| TCGA-A6-2674 | TCGA | 0.74 | low |
| TCGA-AA-3531 | TCGA | 0.83 | low |
| TCGA-AA-A01R | TCGA | 1.46 | high |
| TCGA-AA-3680 | TCGA | 1.01 | high |
| TCGA-G4-6294 | TCGA | 1.12 | high |
| TCGA-AA-3862 | TCGA | 0.82 | low |
| TCGA-CM-6680 | TCGA | 1.05 | high |
| TCGA-G4-6320 | TCGA | 1.63 | high |
| TCGA-AA-A02K | TCGA | 1.56 | high |
| TCGA-CK-6747 | TCGA | 0.35 | low |
| TCGA-G4-6314 | TCGA | 1.01 | high |
| TCGA-A6-A567 | TCGA | 0.54 | low |
| TCGA-D5-6923 | TCGA | 0.97 | high |
| TCGA-G4-6586 | TCGA | 0.28 | low |
| TCGA-D5-6538 | TCGA | 1.58 | high |
| TCGA-AA-A00N | TCGA | 1.55 | high |
| TCGA-AA-3970 | TCGA | -0.04 | low |
| TCGA-CM-6171 | TCGA | 0.26 | low |
| TCGA-F4-6856 | TCGA | 0.05 | low |
| TCGA-A6-4107 | TCGA | 0.77 | low |
| TCGA-AA-A00K | TCGA | 0.94 | low |
| TCGA-DM-A0X9 | TCGA | 0.23 | low |
| TCGA-AA-3556 | TCGA | 0.00 | low |
| TCGA-A6-4105 | TCGA | -0.01 | low |
| TCGA-AA-3968 | TCGA | 1.59 | high |
| GSM2772155 | GEO | 0.68 | low |
| GSM2772185 | GEO | 1.28 | high |
| GSM2772161 | GEO | 0.68 | low |
| GSM2772166 | GEO | 0.85 | low |
| GSM2772177 | GEO | 0.61 | low |
| GSM2772176 | GEO | 0.39 | low |
| GSM2772165 | GEO | 1.55 | high |
| GSM2772147 | GEO | 1.64 | high |
| GSM2772146 | GEO | 0.55 | low |
| GSM2772204 | GEO | 0.61 | low |
| GSM2772191 | GEO | 1.15 | high |
| GSM2772170 | GEO | 1.30 | high |
| GSM2772175 | GEO | 1.45 | high |
| GSM2772157 | GEO | 0.85 | low |
| GSM2772171 | GEO | 1.21 | high |
| GSM2772184 | GEO | 0.94 | low |
| GSM2772241 | GEO | 0.27 | low |
| GSM2772141 | GEO | 0.39 | low |
| GSM2772128 | GEO | 1.51 | high |
| GSM2772263 | GEO | 0.72 | low |
| GSM2772149 | GEO | 0.71 | low |
| GSM2772125 | GEO | -0.08 | low |
| GSM2772150 | GEO | 0.98 | high |
| GSM2772273 | GEO | 0.72 | low |
| GSM2772139 | GEO | 1.35 | high |
| GSM2772197 | GEO | 1.43 | high |
| GSM2772214 | GEO | 1.18 | high |
| GSM2772154 | GEO | 0.73 | low |
| GSM2772145 | GEO | 1.16 | high |
| GSM2772220 | GEO | 0.39 | low |
| GSM2772277 | GEO | 0.22 | low |
| GSM2772142 | GEO | 0.41 | low |
| GSM2772265 | GEO | 1.89 | high |
| GSM2772242 | GEO | 1.52 | high |
| GSM2772266 | GEO | 0.44 | low |
| GSM2772180 | GEO | 0.71 | low |
| GSM2772255 | GEO | 0.44 | low |
| GSM2772268 | GEO | 1.09 | high |
| GSM2772123 | GEO | 0.71 | low |
| GSM2772261 | GEO | 0.78 | low |
| GSM2772205 | GEO | 0.97 | high |
| GSM2772272 | GEO | 0.75 | low |
| GSM2772207 | GEO | 0.24 | low |
| GSM2772169 | GEO | 1.21 | high |
| GSM2772151 | GEO | 0.11 | low |
| GSM2772264 | GEO | 1.07 | high |
| GSM2772174 | GEO | 0.78 | low |
| GSM2772179 | GEO | 1.13 | high |
| GSM2772259 | GEO | 0.77 | low |
| GSM2772156 | GEO | 0.00 | low |
| GSM2772267 | GEO | 1.09 | high |
| GSM2772172 | GEO | 1.37 | high |
| GSM2772187 | GEO | 0.43 | low |
| GSM2772178 | GEO | 1.27 | high |
| GSM2772190 | GEO | 0.96 | low |
| GSM2772158 | GEO | 1.03 | high |
| GSM2772253 | GEO | 1.39 | high |
| GSM2772164 | GEO | 1.64 | high |
| GSM2772160 | GEO | 0.33 | low |
| GSM2772202 | GEO | 0.72 | low |
| GSM2772173 | GEO | 1.88 | high |
| GSM2772167 | GEO | 0.94 | low |
| GSM2772159 | GEO | 1.04 | high |
| GSM2772168 | GEO | 0.59 | low |
| GSM2772237 | GEO | 0.65 | low |
| GSM2772260 | GEO | 0.68 | low |
| GSM2772162 | GEO | 0.29 | low |
| GSM2772135 | GEO | 0.57 | low |
| GSM2772249 | GEO | 0.12 | low |
| GSM2772251 | GEO | 1.11 | high |
| GSM2772252 | GEO | 0.71 | low |
| GSM2772250 | GEO | -0.21 | low |
| GSM2772152 | GEO | 0.77 | low |
| GSM2772246 | GEO | 0.54 | low |
| GSM2772218 | GEO | 0.66 | low |
| GSM2772247 | GEO | 0.95 | low |
| GSM2772206 | GEO | 0.87 | low |
| GSM2772148 | GEO | 0.82 | low |
| GSM2772219 | GEO | 0.68 | low |
| GSM2772153 | GEO | 0.05 | low |
| GSM2772245 | GEO | 1.17 | high |
| GSM2772196 | GEO | 0.72 | low |
| GSM2772230 | GEO | 0.19 | low |
| GSM2772243 | GEO | 1.12 | high |
| GSM2772238 | GEO | 0.58 | low |
| GSM2772194 | GEO | 0.92 | low |
| GSM2772129 | GEO | 1.36 | high |
| GSM2772193 | GEO | 0.20 | low |
| GSM2772229 | GEO | 0.82 | low |

Table.S3.GO-related GSEA between HRG and LRG

| GO.Terms | *p*-value | Adjusted *p* | Enrichment score |
| --- | --- | --- | --- |
| CORNIFIED ENVELOPE | ＜0.001 | 0.03 | -2.24 |
| PEPTIDE CROSS LINKING | ＜0.001 | 0.03 | -2.21 |
| OLFACTORY RECEPTOR ACTIVITY | ＜0.001 | 0.03 | -2.10 |
| SENSORY PERCEPTION OF SMELL | ＜0.001 | 0.03 | -2.10 |
| DETECTION OF STIMULUS INVOLVED IN SENSORY PERCEPTION | ＜0.001 | 0.03 | -2.09 |
| SENSORY PERCEPTION OF CHEMICAL STIMULUS | ＜0.001 | 0.03 | -2.08 |
| ADAPTIVE IMMUNE RESPONSE | ＜0.001 | 0.03 | -1.99 |
| REGULATION OF NATURAL KILLER CELL MEDIATED IMMUNITY | ＜0.001 | 0.03 | -1.98 |
| CELL KILLING | ＜0.001 | 0.03 | -1.97 |
| LYMPHOCYTE CHEMOTAXIS | ＜0.001 | 0.03 | -1.95 |
| DEFENSE RESPONSE TO OTHER ORGANISM | ＜0.001 | 0.03 | -1.94 |
| NATURAL KILLER CELL ACTIVATION | ＜0.001 | 0.04 | -1.91 |
| KILLING OF CELLS OF OTHER ORGANISM | ＜0.001 | 0.03 | -1.91 |
| REGULATION OF CELL KILLING | ＜0.001 | 0.04 | -1.91 |
| LEUKOCYTE PROLIFERATION | ＜0.001 | 0.03 | -1.90 |
| LYMPHOCYTE MIGRATION | ＜0.001 | 0.03 | -1.90 |
| T CELL ACTIVATION | ＜0.001 | 0.03 | -1.89 |
| POSITIVE REGULATION OF CYTOKINESIS | ＜0.001 | 0.04 | -1.88 |
| T CELL ACTIVATION INVOLVED IN IMMUNE RESPONSE | ＜0.001 | 0.03 | -1.88 |
| RESPONSE TO MOLECULE OF BACTERIAL ORIGIN | ＜0.001 | 0.03 | -1.86 |
| NEUTROPHIL MIGRATION | ＜0.001 | 0.03 | -1.86 |
| RESPONSE TO CHEMOKINE | ＜0.001 | 0.04 | -1.86 |
| DEFENSE RESPONSE TO VIRUS | ＜0.001 | 0.03 | -1.84 |
| GRANULOCYTE MIGRATION | ＜0.001 | 0.03 | -1.84 |
| DEFENSE RESPONSE TO BACTERIUM | ＜0.001 | 0.03 | -1.83 |
| POSITIVE REGULATION OF LEUKOCYTE PROLIFERATION | ＜0.001 | 0.03 | -1.82 |
| KERATINIZATION | ＜0.001 | 0.03 | -1.81 |
| B CELL PROLIFERATION | ＜0.001 | 0.04 | -1.81 |
| LYMPHOCYTE DIFFERENTIATION | ＜0.001 | 0.03 | -1.81 |
| EPIDERMAL CELL DIFFERENTIATION | ＜0.001 | 0.03 | -1.80 |
| LYMPHOCYTE ACTIVATION INVOLVED IN IMMUNE RESPONSE | ＜0.001 | 0.03 | -1.79 |
| REGULATION OF LYMPHOCYTE MEDIATED IMMUNITY | ＜0.001 | 0.04 | -1.76 |
| RESPONSE TO VIRUS | ＜0.001 | 0.03 | -1.75 |
| REGULATION OF LEUKOCYTE PROLIFERATION | ＜0.001 | 0.04 | -1.74 |
| POSITIVE REGULATION OF LEUKOCYTE CELL CELL ADHESION | ＜0.001 | 0.04 | -1.73 |
| LYMPHOCYTE MEDIATED IMMUNITY | ＜0.001 | 0.04 | -1.73 |
| HUMORAL IMMUNE RESPONSE | ＜0.001 | 0.04 | -1.72 |
| SKIN DEVELOPMENT | ＜0.001 | 0.03 | -1.70 |
| REGULATION OF T CELL ACTIVATION | ＜0.001 | 0.03 | -1.70 |
| LEUKOCYTE CELL CELL ADHESION | ＜0.001 | 0.04 | -1.67 |
| EPIDERMIS DEVELOPMENT | ＜0.001 | 0.03 | -1.67 |
| LEUKOCYTE DIFFERENTIATION | ＜0.001 | 0.03 | -1.66 |
| POSITIVE REGULATION OF CELL ACTIVATION | ＜0.001 | 0.03 | -1.66 |
| POSITIVE REGULATION OF CYTOKINE PRODUCTION | ＜0.001 | 0.04 | -1.63 |
| REGULATION OF LYMPHOCYTE ACTIVATION | ＜0.001 | 0.04 | -1.61 |
| REGULATION OF IMMUNE EFFECTOR PROCESS | ＜0.001 | 0.04 | -1.55 |
| LEUKOCYTE MIGRATION | ＜0.001 | 0.04 | -1.55 |
